# A Single-Stranded DNA Aptamer That Selectively Binds to *Staphylococcus aureus* Enterotoxin B

**DOI:** 10.1371/journal.pone.0033410

**Published:** 2012-03-16

**Authors:** Jeffrey A. DeGrasse

**Affiliations:** Spectroscopy and Mass Spectrometry Branch, Division of Analytical Chemistry, Center for Food Safety and Applied Nutrition, US Food and Drug Administration, College Park, Maryland, United States of America; University of Houston, United States of America

## Abstract

The bacterium *Staphylococcus aureus* is a common foodborne pathogen capable of secreting a cocktail of small, stable, and strain-specific, staphylococcal enterotoxins (SEs). Staphylococcal food poisoning (SFP) results when improperly handled food contaminated with SEs is consumed. Gastrointestinal symptoms of SFP include emesis, diarrhea and severe abdominal pain, which manifest within hours of ingesting contaminated food. Immuno-affinity based methods directly detect, identify, and quantify several SEs within a food or clinical sample. However, the success of these assays depends upon the availability of a monoclonal antibody, the development of which is non-trivial and costly. The current scope of the available immuno-affinity based methods is limited to the classical SEs and does not encompass all of the known or emergent SEs. In contrast to antibodies, aptamers are short nucleic acids that exhibit high affinity and specificity for their targets without the high-costs and ethical concerns of animal husbandry. Further, researchers may choose to freely distribute aptamers and develop assays without the proprietary issues that increase the per-sample cost of immuno-affinity assays. This study describes a novel aptamer, selected *in vitro*, with affinity to staphylococcal enterotoxin B (SEB) that may be used in lieu of antibodies in SE detection assays. The aptamer, designated APT^SEB1^, successfully isolates SEB from a complex mixture of SEs with extremely high discrimination. This work sets the foundation for future aptamer and assay development towards the entire family of SEs. The rapid, robust, and low-cost identification and quantification of all of the SEs in *S. aureus* contaminated food is essential for food safety and epidemiological efforts. An *in vitro* generated library of SE aptamers could potentially allow for the comprehensive and cost-effective analysis of food samples that immuno-affinity assays currently cannot provide.

## Introduction

Each year, 1 in 6 Americans contract a foodborne disease, and one of the common foodborne bacterial pathogens is *Staphylococcus aureus*, which is estimated to cause 250,000 cases of foodborne illness each year [1]. Enterotoxigenic strains of *S. aureus* secrete a family of small (26–30 kDa) heat-resistant toxins, known as staphylococcal enterotoxins (SEs) [2], [3]. Consumption of improperly handled food contaminated with SEs results in the acute gastroenteritis known as staphylococcal food poisoning (SFP) [3], [4]. The ingestion of as little as 100 ng of SE is sufficient to cause SFP in children, and vulnerable populations can contract SFP with a few micrograms of toxin [5], [6]. Symptoms of SFP include nausea, vomiting, and diarrhea that manifest within 2–6 hours post ingestion and usually subside within 24 hours [7]–[9]. However, in rare cases, the superantigenic SEs can cause symptoms of severe allergic and auto-immune response, as well as toxic-shock syndrome [10]. For all these reasons, SEs pose not only a threat to food safety, but also a food security threat if SEs are produced in a purified form that can be used as deliberate adulterants [11]–[13].

Strains of *Staphylococcus aureus* secrete a closely-related family of 23 SEs (SEA – SE*l*V) [14]. Of these superantigens, only a subset of SEs (SEA-SEI, SER, SES, and SET) are known to cause gastroenteritis, with SEA-SED being the most prevalent enterotoxins found in contaminated food [8], [15], [16].

To detect and quantify SEs in food matrices, immuno-affinity based methods, such as the bead-based multiplexing immuno-affinity assay (Luminex) [17] and lateral flow devices [18], [19], are available; many of these assays have detection limits in the low ppb range. Enzyme-linked immunosorbent assays (ELISA) are commercially available and commonly used in the laboratory [20]–[23]. Various immuno-affinity based biosensors have recently been developed to detect SEs in foods [24], [25]. One such sensor, surface plasmon resonance (SPR), has successfully been used to detect low levels of SEs in assorted food matrices [26]–[28].

However, immuno-affinity based detection of the SEs in food matrices is limited by the availability and quality of antibodies. Antibodies are costly and time consuming to produce and are commonly harvested from mice, sheep and rabbits. The cost of antibody development is reflected in the relatively high cost of commercial immuno-affinity assays. Further, at least two non-classical SEs (SES and SET) have emerged as potent SFP-causing toxins, for which no antibodies or assays are currently available [16].

An aptamer is a nucleic acid (or peptide) that binds to a target with high affinity and specificity [29]. Aptamers are selected *in vitro* from a library of nucleic acids (typically single-stranded DNA or RNA) containing ∼10^15^ individual sequences using a method known as SELEX (systematic evolution of ligands by exponential enrichment) [30]–[32]. SELEX may be accomplished by a number of techniques, one of which involves immobilizing the target onto magnetic beads [33], [34].

Aptamers offer several significant advantages that make them ideal candidates to supplant antibodies for use in toxin detection assays [35]. First, aptamers are discovered *in vitro* which allows any target to be used, despite its toxicity to animals. Second, polymerase chain reaction (PCR) can produce a large, highly pure, quantity of a known aptamer at a relatively low cost. Third, nucleic acids may be modified with a number of functional groups with greater ease, and without negative effects (e.g. loss of affinity), than an antibody. Finally, aptamers are inherently more stable over a greater range of conditions than antibodies. Indeed, many immuno-affinity assays have been successfully transferred to aptamer-affinity assays with similar figures of merit [36].

Recently, aptamers with affinity to toxins and whole-cell pathogens important to the field of food safety have been successfully discovered [37]–[39]. A single-stranded DNA (ssDNA) aptamer to SEB was first described by Bruno, *et. al.* [40]. Unfortunately, the sequence of that aptamer has not been disclosed, which severely limits its potential utility for the protection of public health. Soon after, Purschke, *et. al.*, discovered a Spiegelmer (an enantiomeric _L_-DNA) ssDNA aptamer with affinity to SEB [41]. While the Spiegelmer has a promising application with respect to therapeutics and drug design, the fact the one cannot easily amplify a Spiegelmer by PCR hinders its use in general aptamer-affinity assays. Neither the Bruno nor the Purschke aptamers were demonstrated to be selective for SEB relative to other *Staphylococcal* enterotoxins.

This paper serves to outline the general and rapid method that was used to discover an aptamer with affinity to SEB [42]. Further, using aptamer-precipitation experiments, the aptamer APT^SEB1^ was characterized to bind to SEB with high selectivity amongst other enterotoxins. This protocol will serve the future of the aptamer initiative at the US FDA and be applied to target molecules of interest to food safety such as toxins, allergens and even entire pathogens.

## Materials and Methods

The SELEX methodology outlined below is adapted from the work of Murphy, *et. al.* [43].

### Preparation of nucleic acids

The DNA sequences used in this work are listed in Table 1. The library template (APT^LIB^) consists of a central string of 40 randomized nucleotides that are flanked by defined primer binding regions necessary for PCR amplification. The forward and reverse primers (with and without a biotin moiety attached to the 5′ nucleotide) were synthesized at 25 nmole scale and then desalted (Integrated DNA Technologies [IDT], Coralville, IA). The APT^LIB^ was synthesized at 1 µmole scale, with machine mixing for bases within the center random sequence domain, and purified by PAGE (IDT). All DNA stock was maintained at 100 µM in 10 mM Tris, pH 8.0, 0.1 M EDTA, and stored at −40°C until use.

**Table 1:** The primer and library sequences used in this study as well as the sequences of the two aptamers discovered in this work. APT^SEB1^ was reported in 48 out of 49 sequences.

Forward Primer	5′-GGT ATT GAG GGT CGC ATC
Reverse Primer	5′-AGA GGA GAG TTA GAG CCA TC
APT^LIB^	5′-GGT ATT GAG GGT CGC ATC-N_40_-GAT GGC TCT AAC TCT CCT CT
APT^SEB1^	5′-GGT ATT GAG GGT CGC ATC **CAC TGG TCG TTG TTG TCT GTT GTC TGT TAT GTT GTT TCG T**GA TGG CTC TAA CTC TCC TCT
APT^SEB2^	5′-GGT ATT GAG GGT CGC ATC **CCG TAG TGT GTT CTT ATT CGT GTC TGT GTG TGT TCT GTC G**GA TGG CTC TAA CTC TCC TCT

### Preparation of coated magnetic beads

Thirty micrograms of highly purified staphylococcal enterotoxin B (SEB, Toxin Technology, Sarasota, FL) was bound to 2×10^8^ (3 mg) Dynabeads© M-270 Epoxy using the Dynabeads Co-Immunoprecipitation Kit (Life Technologies, Grand Island, NY). Following conjugation and washing, the SEB-coated magnetic beads were suspended at 6.7×10^5^ beads/µl, or 10 µg/µl, in PBS-T (10 mM phosphate buffer, 2.7 mM KCl, 140 mM NaCl, 0.05% Tween, pH 7.4, Sigma, St. Louis, MO). Uncoated beads for counter-selection were produced in a similar manner without a ligand.

Mass spectrometry was used to confirm the binding of SEB to the Dynabeads. The detailed protocol is described elsewhere [44]. Briefly, 2.7×10^7^ coated-beads were washed and resuspended in 50 µl of 50 mM ammonium bicarbonate and 9% acetonitrile. One microgram of the protease trypsin was added to the suspended beads and the mixture was allowed to react at 60°C for 4 hours before quenching the proteolysis with 1% acetic acid (final concentration). The resultant peptides were analyzed by LC-MS (LTQ, Thermo Fisher Scientific, Waltham, MA) and those data were compared to a reference standard SEB (10 ng/µl in of 50 mM ammonium bicarbonate and 9% acetonitrile) that was treated in the same manner.

### SELEX

For the first round of SELEX, 5 nmoles (potentially ∼3×10^15^ different ssDNA sequences) of the library were diluted 10-fold in PBS-T. The ssDNA was denatured at 95°C for 5 minutes and then left to cool on ice for at least 10 minutes. Fifty microliters (3.4×10^7^) of beads were washed twice in 500 µl PBS-T and then resuspended in 1 ml PBS-T. The ssDNA library and beads were diluted into 48.5 ml PBS-T in a 50 ml centrifuge tube. To limit non-specific interactions, 50 µl of 1 mg/ml BSA (Sigma) and 5 µl of 1 mg/ml poly(deoxyinosinic-deoxycytidylic) acid (Sigma) were also added. The mixture was allowed to incubate, with gentle rotation, for 30 minutes.

To double the number of ssDNA sequences that are exposed to the SEB-coated beads, two incubations were carried out in parallel for a total of ∼6×10^15^ unique ssDNA sequences. In practice, several parallel 50 ml reactions can be accomplished simultaneously, if one desires a larger library.

The centrifuge tubes were placed onto a large magnet (DynaMag™-50 Magnet, Life Technologies) for 20 minutes to collect the magnetic beads. The majority of the supernatant was aspirated and the multiple reactions were pooled. After aspirating all excess supernatant, the tube was removed from the magnet and the beads were resuspended prior to transferring the mixture to a microcentrifuge tube. The microcentrifuge tube was placed onto a smaller magnet (DynaMag™-2 Magnet, Life Technologies). After removing the supernatant, the beads were washed once with 500 µl of PBS-T, which was immediately removed.

All of the beads were transferred to a PCR tube using 22.5 µl of nuclease-free water. To the bead and water mixture, 2.5 µl of 10 µM forward and 5′ biotinylated reverse primer mix and 25 µl of AmpliTaq Gold® Fast PCR Master Mix (Life Technologies) was added. To produce a significant amount of dsDNA while reducing the possibility of incorrectly-sized products, multiple PCRs were carried out in tandem.

The first PCR (PCR_1_) proceeded as follows: 10 minutes at 95°C followed by 15 cycles of 96°C for 3 seconds, 56°C for 3 seconds, and 68°C for 5 seconds. After the final cycle the reaction was held at 72°C for 10 seconds before cooling the PCR_1_ product to 4°C. The paramagnetic beads were removed via magnet from the PCR_1_ product.

The second PCR (PCR_2_) proceeded with 4 reaction tubes each using PCR_1_ product as the template. One microliter of PCR_1_ product was added to 21.5 µl of nuclease-free water, 2.5 µl forward and 5′ biotinylated reverse primer mix and 25 µl of AmpliTaq Gold® Fast PCR Master Mix. PCR_2_ proceeded as follows: 10 minutes at 95°C followed by 35 cycles of 96°C for 3 seconds, 56°C for 3 seconds, and 68°C for 5 seconds. After the final cycle the reaction was held at 72°C for 10 seconds before cooling the PCR_2_ product to 4°C.

At this point, the 4 PCR_2_ products were pooled into one vial. The quality of PCR products was monitored by E-Gel® 4% high-resolution agarose (Life Technologies). Twenty microliters of PCR_2_ product was loaded onto the gel and visualized by ethidium bromide staining. The pooled PCR product (135 µl) was mixed with 34.5 µl of 5 M NaCl and then incubated with 1 mg of Dynabeads® M-270 Streptavidin (Life Technologies) for 10 minutes. To separate the ssDNA aptamer candidates from the complementary strand, the beads were incubated for 5 minutes in 50 µl of freshly prepared (daily from a 1 M NaOH stock stored at 4°C) 100 mM NaOH. To adjust the pH to 7.4, the supernatant was transferred to a microcentrifuge tube containing 850 µl PBS-T and 100 µl sodium phosphate monobasic.

The ssDNA was denatured at 95°C for 5 minutes and then cooled on ice before proceeding to the next round. Table 2 contains the number of beads and incubation times used for rounds 2–14.

**Table 2:** Values for the number of beads and incubation times used in each round.

Round	Counter Selection Beads	Selection Beads	Incubation Time (min)
1	0	3.4×10^7^	30
2–3	0	1.3×10^7^	10
4–6	3.4×10^7^	6.7×10^6^	10
7–9	3.4×10^7^	2.0×10^6^	10
10–11	3.4×10^7^	6.7×10^5^	10
12–14	3.4×10^7^	6.7×10^5^	0

The values were modulated in later rounds to increase the stringency of the SELEX protocol.

In the counter-selection rounds (4–14), the cooled ssDNA was first incubated with counter-selection beads for 10 minutes. Then, using a magnet, the supernatant was transferred to the appropriate amount of washed selection beads to incubate (see Table 2).

After round 14, the PCR product was inserted into the TOPO® TA cloning vector (Life Technologies) according to manufacturer instructions. The vector then was inserted into One Shot® Top10 *E. coli* (Life Technologies) using the rapid chemical transformation protocol. The *E. coli* (50 µl) was plated and grown overnight on pre-warmed (37°C) LB agar plates containing 100 µg/ml ampicillin. The plates with a few hundred colonies were sent to GENEWIZ (South Plainfield, NJ) where 50 colonies were randomly selected for Sanger sequencing using the T7 sequencing primer that was incorporated into the TOPO TA vector.

The sequences were trimmed to remove known plasmid and primer regions, assessed for quality (i.e. proper length and sequence confidence), and then aligned with Geneious 5.5 [45] and ClustalW2 [46].

### Aptamer-precipitation assay – BSA∶SEB 10∶1

In separate tubes, 5 µg of biotinylated (1) APT^SEB1^, (2) three random 78 base ssDNA molecules, and (3) forward primer were diluted into 200 µl of PBS-T. A sixth tube contained no DNA and served as a negative control. The three random 78 base ssDNA molecules were identified in other SELEX experiments and were not predicted to exhibit affinity towards SEB. The diluted ssDNA was heated to 95°C for 5 minutes and then placed on ice. Meanwhile, 2 mg of Dynabeads M-270 Streptavidin were aliquoted into 6 tubes. The beads were washed twice with 500 µl of PBS-T.

After the final wash was removed, the chilled DNA was added to the streptavidin-coated magnetic beads. The beads and DNA were mixed by rotation at room temperature for 30 minutes. The beads were then washed 3 times with 500 µl PBS-T and then resuspended in 500 µl PBS-T containing 10 µg BSA. Following a thorough resuspension, 1 µg of SEB was added to each of the 5 tubes containing DNA-coated beads and the negative control. The beads were allowed to incubate in the protein mixture for 30 minutes at room temperature with rotation.

Afterwards, the beads were again washed 3 times with 500 µl PBS-T. After the final wash was removed, 50 µl of 1X LDS sample buffer (Life Technologies) supplemented with 0.5 M NaCl was added on top of the coated beads, and the mixture was incubated at 50°C with agitation for 10 minutes. For a positive control, standards of BSA and SEB (100 ng, each) were diluted into 50 µl of 1X LDS loading buffer (Life Technologies) supplemented with 0.5 M NaCl.

Twenty five microliters each of the samples and standards, as well as 5 µl of the molecular weight ladder (SeeBlue Plus2 Pre-Stained Standard, Life Technologies), were loaded onto a NuPAGE® 4–12% Bis-Tris pre-cast polyacryamide gel (Life Technologies) with MOPS as the running buffer. Electrophoresis was conducted at 125 V for the initial 5 minutes and then at 200 V for approximately 30 minutes. The proteins were visualized with silver stain (Thermo Fisher Scientific).

### Aptamer-precipitation assay – a mixture of closely-related enterotoxins

In two tubes, 5 µg of biotinylated APT^SEB1^ was conjugated to 2 mg of streptavidin Dynabeads, as outlined above. Following several washes with PBS-T, the aptamer coated beads were incubated at room temperature for 30 minutes with one of two mixtures. Mixture 1 contained the following enterotoxins (1 ng/µl): SEA, SEB, SEC_1_, SEC_2_, SEC_3_, SED, and SEE. Mixture 2 contained the same enterotoxins as mixture 1, but without SEB. The total volume of reaction was 1 ml. The coated beads were then washed, and the toxins were eluted and analyzed by PAGE, as described above.

### Aptamer-precipitation assay – *S. aureus* culture supernatants

Five micrograms of biotinylated APT^SEB1^ was prepared and conjugated to 2 mg streptavidin Dynabeads as described above. The washed APT^SEB1^-coated beads were incubated for 30 minutes at room temperature with 3 ml of cell-free culture supernatant (CFCS) from the following *S. aureus* strains: BAA1747 [47] (ATCC, Manassas, VA), BAA1751 (ATCC), NRS109 [48] (NARSA, Chantilly, VA), and NRS111 [49] (NARSA). The beads were subsequently washed 3 times with 500 µl PBS-T and prepared for SDS-PAGE analysis as described above.

## Results and Discussion

### A generalized ligand immobilization procedure

Intact, unmodified, natively-folded SEB was directly immobilized to M-270 epoxy Dynabeads using already established conjugation strategies. This method was chosen over various other methods because the epoxy-functionalized surface allows for a generalized conjugation protocol to bind proteinacious ligands to the surface of the bead without prior protein modification (i.e. biotinylation). Uncoated beads were generated using the same protocol, but without a protein ligand. These beads were used in the “counter-selection” steps outlined below in order to remove ssDNA aptamer candidates that may have non-specifically bound to the unfunctionalized regions of the bead surface during SELEX. Mass spectrometry was used to ensure that the conjugation reaction yielded SEB covalently bound to beads prior to SELEX (data not shown). Efforts were not made to calculate the concentration of the bound SEB because one could simply modulate the number of beads used for a round of selection to increase selective pressure.

### *In vitro* enrichment of ssDNA that binds to SEB

Many SELEX protocols are quite labor intensive or require specialized equipment such as microfluidics [35]. This work was a refinement of the SELEX protocol developed by Murphy, et. al., and allowed for the rapid discovery of high affinity ssDNA aptamers [43]. Here, SELEX began with a random library of 6×10^15^ unique DNA sequences. The first round of selection was the most liberal with respect to selective pressure. The entire DNA sequence space was exposed to 3.4×10^7^ beads and had the longest incubation time at 30 minutes (see Table 2). Throughout SELEX, the DNA product from a round of selection was analyzed by agarose (4%) gel electrophoresis, and was considered successful when a properly sized DNA band was visualized (data not shown). It was observed that the quality of the DNA band improved during SELEX. The APT^LIB^ ran as a band with a smeared tail, but as SELEX progressed, the DNA collapsed down to a sharp band with a well-defined border, suggesting sequence enrichment of a subset of nucleic acids.

Once multiple copies of each candidate sequence were present after PCR (i.e. after round 1), selective pressure was increased gradually. Increasing the selective pressure forced the *in vitro* selection of the best ssDNA aptamer by eliminating low-affinity or non-specifically binding ssDNA while simultaneously enriching for high quality aptamers. Variables affecting the stringency included increasing the amount of wash steps, decreasing the amount of selection beads, and shorter incubation times (see Table 2).

Beginning at round 4, a counter-selection step was introduced. Counter-selection served to remove those ssDNA sequences that bound directly to the epoxy Dynabead surface (or the vial walls) and not to SEB. Heated and cooled ssDNA were washed over 3.4×10^7^ uncoated beads for 10 minutes. Following incubation, the beads were partitioned using a magnet and the supernatant was immediately added to the specified amount of SEB-coated beads and incubated for the specified time (see Table 2). Remarkably, a significant amount of ssDNA was present after rounds 12 through 14 despite no incubation time, suggesting the enrichment of high affinity ssDNA molecules from the APT^LIB^.

The method was rapid enough to allow for the completion of ∼10 rounds of selection per week. In practice, many targets may be screened at once, as an efficient use of time and resources, to produce a suite of aptamers.

### Sequence analysis of aptamer candidates

After round 14, the PCR_2_ product was inserted into the TOPO TA cloning vector. This cloning strategy was selected because it did not require the use of restriction enzymes as this technology exploits the 3′ adenosine overhangs that result from Taq polymerase chain extension. Thus the PCR product can be directly inserted into the cloning vector.

After transforming the loaded vector into *E. coli*, the bacteria were plated onto ampicillin selective plates. Fifty positive clonal colonies were sequenced. Of the 49 sequences returned, 48 were identical (see Table 1). APT^SEB1^ was reported as 5′-GGT ATT GAG GGT CGC ATC CAC TGG TCG TTG TTG TCT GTT GTC TGT TAT GTT GTT TCG TGA TGG CTC TAA CTC TCC TCT. As an example of the stringent selection, APT^SEB1^ and APT^SEB2^ are 76% identical over a local 25 nucleotide region, and 50% identical across the entire sequence. That APT^SEB2^ is similar to APT^SEB1^ demonstrates the high degree of selective enrichment of APT^SEB1^ from the original random 6×10^15^ unique sequences.

### Aptamer-precipitation assay

**Figure 1:**
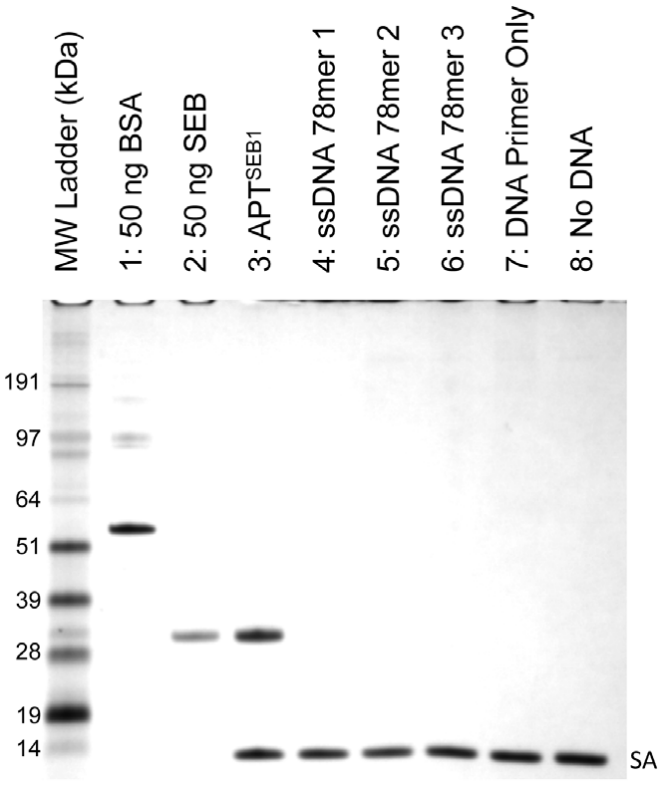
APT^SEB1^ binds to SEB, but not BSA. Aptamer-precipitation of SEB from 10-fold excess of BSA using several DNA sequences was visualized by 4–12% SDS-PAGE with silver stain. Dynabeads® M-270 Streptavidin magnetic beads coated with APT^SEB1^ (lane 3), random 78-base ssDNA (lanes 4–6), PCR forward primer (used in this study, lane 7), and nothing (lane 8), were incubated in 500 µl PBS-T incurred with 10 µg BSA and 1 µg SEB. After washing the Dynabeads with PBS-T, the protein eluate (lanes 3–8) was loaded onto the SDS-PAGE gel. Lanes 1 and 2 contain 50 ng of standard BSA and SEB, respectively. The protein bands labeled as “SA” represent the monomer of streptavidin liberated by the elution protocol.

APT^SEB1^ was chosen for further characterization due to its over-representation relative to APT^SEB2^. The aptamer was synthesized (IDT) with a 5′ biotin moiety to allow for easy attachment to streptavidin coated Dynabeads. Once conjugated, the coated beads were used for an aptamer-precipitation assay to partition SEB from a 10-fold excess solution of BSA. Following incubation, the beads were extensively washed with PBS-T to fully remove non-specifically bound proteins (i.e. BSA). Relative to the negative controls (Figure 1, lanes 4–8), APT^SEB1^ selectively partitioned SEB from BSA (Figure 1, lane 3). That the SEB was not fully removed from the aptamer-coated beads despite aggressive wash steps suggested an affinity sufficient for successful aptamer-precipitation of SEB from a sample matrix.

**Figure 2:**
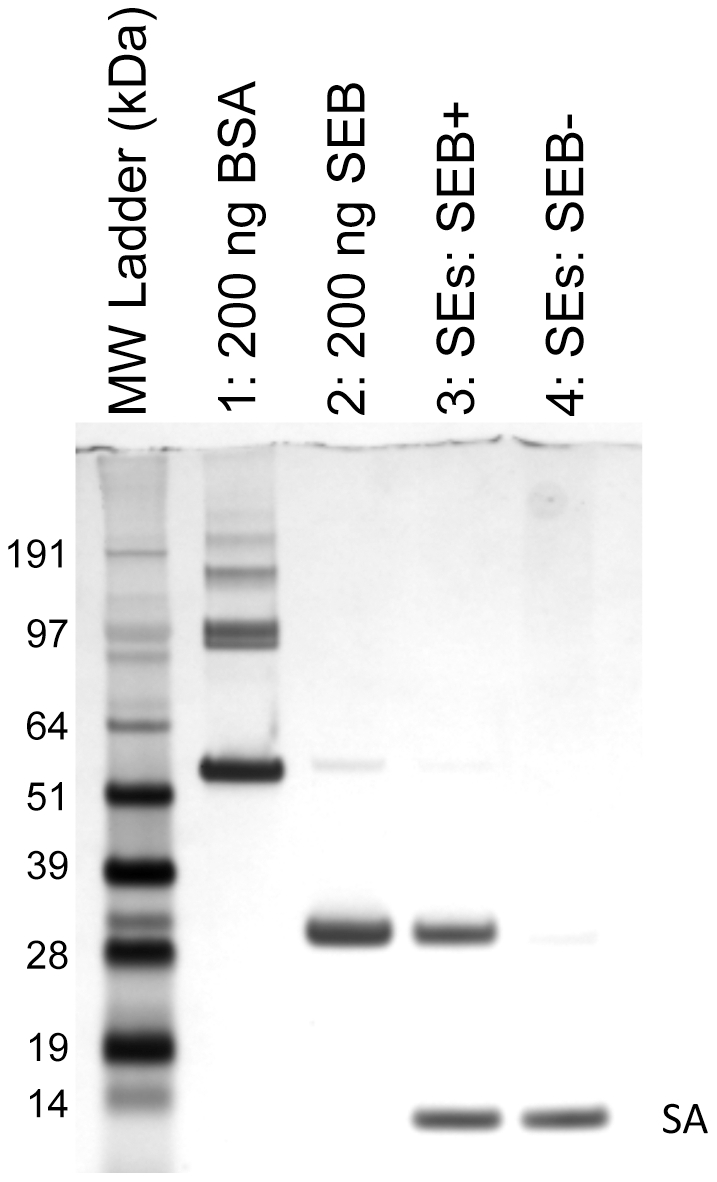
APT^SEB1^ is selective for SEB but not other closely related enterotoxins. Aptamer-precipitation of SEB from a mixture of enterotoxins was visualized by 4–12% SDS-PAGE with silver stain. Dynabeads® M-270 Streptavidin magnetic beads coated with APT^SEB1^ were incubated in 1000 µl PBS-T incurred with 1 µg each of SEA, SEC_1_, SEC_2_, SEC_3_, SED, and SEE. The aptamer-precipitation was carried out either with (lane 3) or without (lane 4) 1 µg SEB present in the mixture. After washing the Dynabeads with PBS-T, the protein eluate (lanes 3–8) was loaded onto the SDS-PAGE gel. Lanes 1 and 2 contain 200 ng of standard BSA and SEB, respectively. The protein bands labeled as “SA” represent the monomer of streptavidin liberated by the elution protocol.

To further demonstrate the selectivity of APT^SEB1^, a similar aptamer-precipitation experiment was conducted using a mixture of closely related (relative to primary structure) *Staphylococcal* enterotoxins. As observed in Figure 2, APT^SEB1^ successfully partitioned SEB away from the other classical enterotoxins. Remarkably, even though pair-wise alignment analysis of SEB (GI:15625508) and SEC_1_ (GI:119625) revealed an identity of 68%, APT^SEB1^ did not significantly bind to any of the SEC variants.

Within a complex mixture, affinity and selectivity are two critical requirements of an aptamer if it is to be used in any assay. To further challenge the selectivity of APT^SEB1^, an aptamer-precipitation assay was performed on a complex mixture of toxins. Four strains, for which toxin profiles are known (Sandra Tallent, personal communication), were cultured, and the toxin-rich cell-free culture supernatant (CFCS) was extracted. Strain BAA1747 is known to secrete SEB (along with SEK and SEQ). The other three strains, BAA1751 (SEG, SEI, SEN, SEO, SEU), NRS111 (SEA, SEC_3_, SEE, TSST, SEK, SEL, SEQ), and NRS109 (SEC_2_, SED, SEG, SEI, SEJ, SEL, SEM, SEN, SEO, SER) do not secret SEB; however, together they secrete 17 non-SEB toxins. These 4 strains offered sufficient toxin diversity to challenge the selectivity of APT^SEB1^.

**Figure 3:**
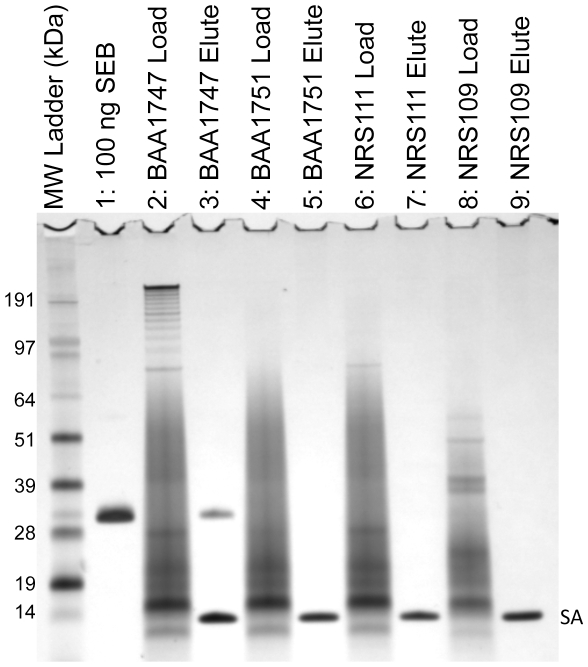
APT^SEB1^ is selective for SEB within a complex mixture. The toxin-rich cell-free culture supernatant from four *S. aureus* strains was assayed for the presence of SEB by aptamer-precipitation. Five microliters of each culture supernatant was loaded onto an 4–12% SDS-PAGE gel to determine the protein content (lanes 2, 4, 6, 8). Three milliliters of each culture supernatant was incubated with APT^SEB1^-coated Dynabeads. After washing with PBS-T, the resultant protein eluate from the APT^SEB1^-coated Dynabeads was analyzed (lanes 3, 5, 7, 9). By PCR and ELISA analysis (Sandra Tallent, personal communication) the four strains potentially express a total of 17 enterotoxins and toxic shock syndrome toxin. However, only strain BAA1747 contains the gene for SEB. The protein bands labeled as “SA” represent the monomer of streptavidin liberated by the elution protocol.

The protein profiles of each CFCS were quite complex and many proteins were not adequately resolved when loaded directly on the polyacrylamide gel (Figure 3, lanes 2, 4, 6, & 8). However, when APT^SEB1^ was incubated with the CFCS from BAA1747, a single protein whose MW is consistent with the SEB standard was isolated (Figure 3, lane 3). SEB, or any other toxin or protein, was not retained by the APT^SEB1^ coated beads from the toxin-rich CFCS of the other three strains (Figure 3, lanes 5, 7, & 9). The results of the aptamer-precipitation experiments suggested that not only does APT^SEB1^ have an affinity to SEB, but it is also highly selective for SEB.

### Perspectives

There is a need for the rapid development and deployment of aptamers with affinity to toxins and allergens related to food safety. Aptamers and their use in aptamer-affinity assays would serve two roles. First, with this modified protocol, aptamers could be efficiently developed to molecules for which there are no available antibodies and immuno-affinity assays. Second, aptamers could supplant commercial antibodies to generate aptamer-affinity assays that are lower in cost and can be widely distributed.

One of the more important advantages of an aptamer over an antibody is the ability to freely distribute the molecule to allow other scientists to immediately use this aptamer to develop an assay that suits their needs. Aptamer-affinity assays could be developed and evaluated in a manner similar to that of immuno-affinity assays. The key difference is that any laboratory with the aptamer sequence could perform the assay.

APT^SEB1^ marks the beginning of the US FDA's aptamer initiative, with respect to food safety and security. Aptamers possess binding properties similar to receptors or antibodies, without the ethical concerns of animal use, and they may be freely distributed throughout the world, making their use in assays an attractive alternative to immuno-affinity assays. Efforts are underway to develop and optimize assays with APT^SEB1^ to detect and quantify the presence of SEB in food matrices. Specifically, work developing an assay utilizing the surface plasmon resonance biosensor platform and other aptamer-affinity assays similar to that used in this study are currently in progress.

Further, selection for a full range of aptamers corresponding to the family of *S. aureus* exotoxins is in progress, and the selected aptamers will be subsequently applied to the developed and optimized assays. With a concerted effort, aptamers could not only reduce the cost of food safety field assays, but also allow for widespread implementation of those assays by local health inspection agencies thereby empowering them with the tools necessary to enhance public health protection.
